# Neighborhood influences on the development of self-regulation among children of color living in historically disinvested neighborhoods: Moderators and mediating mechanisms

**DOI:** 10.3389/fpsyg.2022.953304

**Published:** 2022-10-26

**Authors:** Alexandra Ursache, Rita Gabriela Barajas-Gonzalez, Spring Dawson-McClure

**Affiliations:** Department of Population Health, NYU Grossman School of Medicine, New York, NY, United States

**Keywords:** self-regulation, built environment, social environment, neighborhood, early childhood, structural racism

## Abstract

We present a conceptual model of the ways in which built and social environments shape the development of self-regulation in early childhood. Importantly, in centering children of color growing up in historically disinvested neighborhoods, we first describe how systemic structures of racism and social stratification have shaped neighborhood built and social environment features. We then present evidence linking these neighborhood features to children’s development of self-regulation. Furthermore, we take a multilevel approach to examining three potential pathways linking neighborhood contexts to self-regulation: school environment and resources, home environment and resources, and child health behaviors. Finally, we consider how racial-ethnic-cultural strengths and multilevel interventions have the potential to buffer children’s development of self-regulation in disinvested neighborhood contexts. Advancing multilevel approaches to understand the development of self-regulation among children of color living in historically disinvested neighborhoods is an important step in efforts to promote equity in health and education.

## Introduction

Self-regulation includes processes ranging from automatic to effortful regulation of stress physiology, emotion, attention, and executive function and is an important predictor of children’s learning and academic achievement (Bull and Scerif, 2001; Raver et al., 2007; Morrison et al., 2010; McClelland and Cameron, 2011; Blair et al., 2015; Blair and Raver, 2015; Blair and Ku, 2022). A number of studies support the integration of these components into a hierarchical integrated model of self-regulation which describes reciprocal and recursive relations among genetic, physiological, behavioral, emotional, and executive function components of self-regulation (Blair and Ku, 2022). Importantly, although these components of self-regulation are separable, they are also intricately linked at any given point in time, as well as across development. As such, direct environmental impacts on more automatic aspects of self-regulation, such as stress physiology, will likely also shape more effortful aspects of self-regulation, such as executive functions. We focus on executive function and emotion regulation as two aspects of self-regulation that develop rapidly in early childhood and that underlie multiple aspects of early school success and later academic achievement (Calkins and Marcovitch, 2010). Furthermore, the inclusion of stress physiology within this model of self-regulation highlights the very important ways in which children’s environment can ‘get under the skin’ to shape other aspects of self-regulation.

Although self-regulation is specified as highly dependent on context, past work has largely focused on family level adversity and poverty-related risk. A small literature has begun to investigate self-regulation in neighborhood context. To guide this area of inquiry in the service of creating more equitable opportunities for children to thrive, more work is needed to conceptualize self-regulation as situated in neighborhood contexts (Blair and Ku, 2022). Most work examining relations of neighborhood disadvantage to child development has focused on neighborhood concentration of poverty (Minh et al., 2017). For example, evidence suggests that moving out of high poverty neighborhoods, as compared to remaining in these neighborhoods, is associated with increases in self-regulation (Roy et al., 2014). Composite indices of disadvantage have also been used to demonstrate links to children’s stress physiology (Finegood et al., 2017) and to differences in functioning of brain regions underlying self-regulatory processes (Gard et al., 2021). On the one hand, composite indices or profiles capturing co-occurring risks can be highly useful for comparing across different neighborhoods because they condense the complex exposures encompassed by neighborhoods (Messer et al., 2006; McCoy et al., 2022). On the other hand, however, understanding the ways in which specific aspects of the built and social environments may influence children’s self-regulation can serve to highlight new opportunities for enriching neighborhood contexts to support the development of self-regulation.

**Figure 1:**
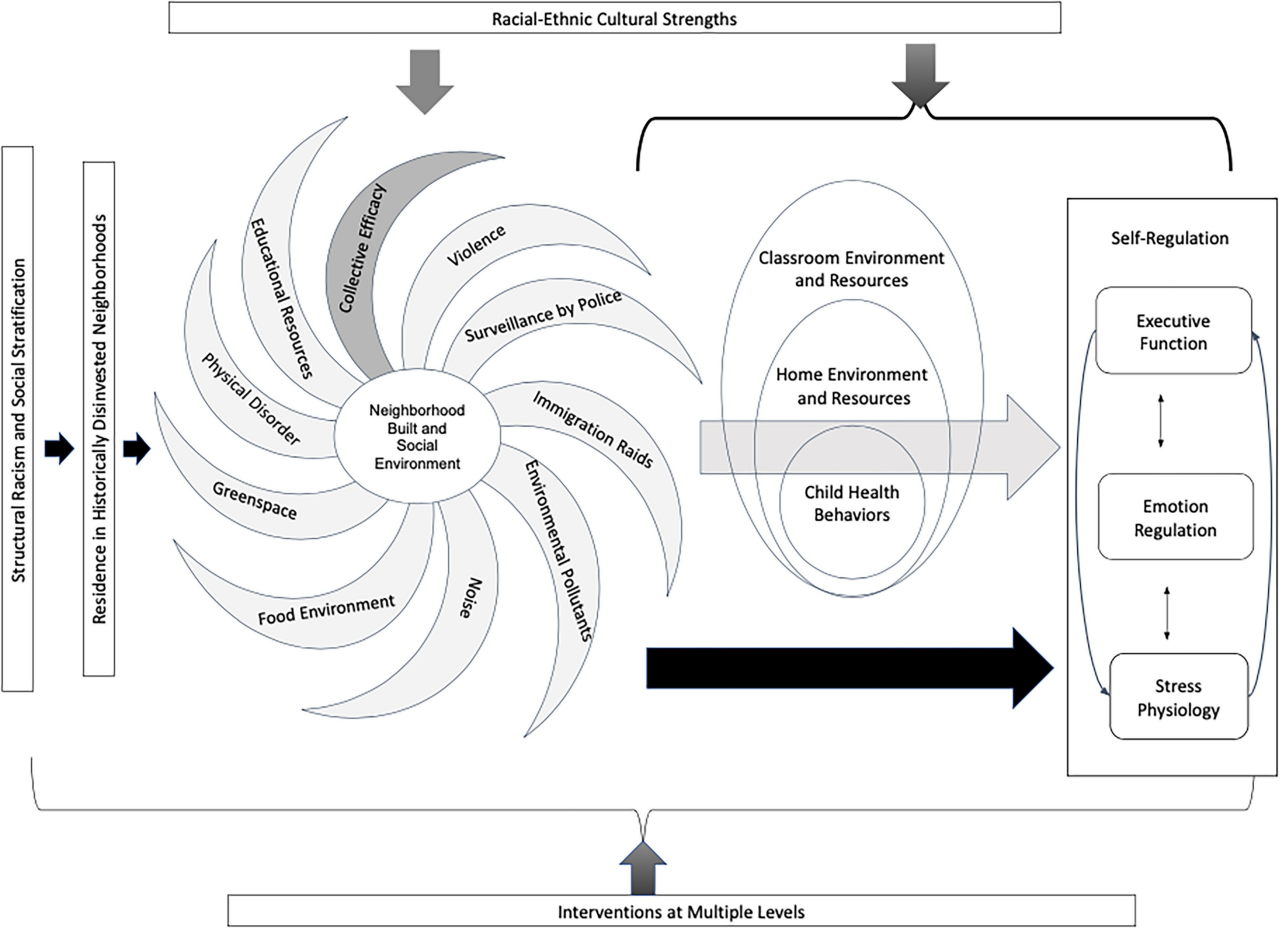
Conceptual model of built and social environment influences on the development of early childhood self-regulation in historically disinvested neighborhoods.

We propose a conceptual model of self-regulation development in neighborhood context which centers children and families of color who are living in historically disinvested neighborhoods (Figure 1). Our model focuses on early childhood as a time of high plasticity in the brain areas underlying self-regulation and rapid development of self-regulatory systems from more automatic to more effortful processes (Carlson et al., 2005; Garon et al., 2008). Our approach draws on theoretical foundations from Garcia-Coll’s integrative model of child development (Garcia-Coll et al., 1996) and Rogers’ M(ai)cro conception of development (Rogers et al., 2021) to emphasize the ways in which the structures of systemic racism and social stratification hinder children’s development. These macro-level forces shape the neighborhoods in which children of color live, children’s exposure to neighborhood built and social environments, and thus their daily experiences which intimately influence the developmental process of self-regulation. Most of the literature to date, however, has not incorporated an adequate examination of these structures and how they create inequality across neighborhoods (Minh et al., 2017). Models that attempt to situate self-regulation in the neighborhood context while largely ignoring structures of historical disinvestment (e.g., Maniar and Zaff, 2011) have the potential to harm communities of color and distort the role of self-regulation in improving health and educational equity. For example, a model that does not mention the enduring impacts of racism but conceptualizes the burden of inequities in underserved neighborhoods as in part resulting from lower self-regulation of individuals (Maniar and Zaff, 2011) may perpetuate a narrative in which communities of color are blamed for the social, economic, and health disparities that they experience.

Drawing on Rogers’ M(ai)cro conception of development (Rogers et al., 2021) we take as our starting point the macrosystem structures of racism and social stratification, which have led to high concentrations of families of color with low incomes living in neighborhoods faced with a number of built and social environment risks. The built and social environment are hypothesized to impact children’s self-regulation development directly and through multilevel mediating pathways including classroom environment and resources, home environment and resources, and children’s health behaviors. Drawing on Garcia-Coll’s integrative model (Garcia-Coll et al., 1996), we emphasize that neighborhoods also consist of internal community resources that can support or hinder child development. Racial-ethnic cultural strengths of families of color may buffer neighborhood risk by promoting neighborhood social support and strengthening protective family processes. Additionally, interventions at multiple ecological levels can support communities in changing neighborhoods or buffering the impacts of neighborhood risk factors on child self-regulation.

## Structural racism and social stratification create historically disinvested neighborhoods

Structural racism and social stratification have influenced the creation of historically disinvested neighborhoods and have led to high concentrations of low-income families of color living in these areas (Cashin, 2004; Faber, 2020). As a prime example, “redlining”—“the practice of denying borrowers access to credit based on the location of properties in minority or economically disadvantaged neighborhoods” led to disinvestment in these neighborhoods (Mitchell and Franco, 2018 p 5). Several studies have utilized the “Residential Security” maps drawn up in the 1930s by the Home Owners’ Loan Corporation, an agency of the federal government, to quantify the enduring impacts of redlining. On the HOLC maps, neighborhoods were rated according to their perceived level of mortgage lending risk. These evaluations were based on a number of characteristics, but a major factor was the race and ethnicity of residents in the neighborhood (Meisenhelter, 2018). Although there is some debate as to the exact use of these maps, these maps clearly represent local-level lending decision makers’ collective understanding about neighborhood risk (Mitchell and Franco, 2018). This conflation of race with lending risk led to a self-reinforcing cycle of beliefs in racial hierarchy, unequal investment in neighborhoods, white families moving to suburbs while Black families remained crowded in inner city areas, and in turn increases in suburban property values coupled with deteriorating city neighborhoods (Faber, 2020). This cycle continues today as homes in white neighborhoods are appraised higher than homes in Black and Latinx neighborhoods, with some estimates indicating about a 20% gap (Perry et al., 2018; National Fair Housing Alliance, 2022).

Today, the majority of areas that were identified as most risky on the HOLC maps remain low-to-moderate income and minority neighborhoods (Mitchell and Franco, 2018), and redlining continues to have enduring effects on these neighborhood environments (Meisenhelter, 2018; Schwartz et al., 2021). For example, over 80 years later, neighborhoods that were redlined have less greenspace (Nardone et al., 2021), greater density of tobacco retailers (Schwartz et al., 2021), are more likely to be “food deserts” with lower access to supermarkets and higher reliance on convenience stores (NYLSRJP, 2013; Zhang and Ghosh, 2016), and have higher rates of gun violence (Jacoby et al., 2018). Furthermore, the enduring impacts of racism can be seen when looking across a broad swath of neighborhood factors that support child health and development. For example, the Child Opportunity Index (COI) is a comprehensive measure that captures 29 neighborhood factors shown to predict child outcomes. In examining Child Opportunity Scores by race across the 100 largest metro areas in the United States, the score for white children was more than three times the score for Black children and more than two times the score for Latinx children (Acevedo-Garcia et al., 2019). Additionally, the HOLC rating of the neighborhood in which children grow up has causal and economically meaningful impacts on their outcomes as adults (e.g., household income, credit scores, and likelihood of living in a high-poverty neighborhood; Aaronson et al., 2021). In addition to redlining, the impacts of systemic racism likely constrain neighborhood residence for families of color in less well documented ways. For example, undocumented immigrants have reported choosing to live in majority Black or Latinx neighborhoods rather than in higher opportunity white neighborhoods because law enforcement patrols in white neighborhoods are perceived to target Latinx individuals, specifically those who are undocumented (Asad and Rosen, 2019). Thus, structural racism and poverty have led to the enduring segregation of children of color into neighborhoods plagued by built and social environment risks.

## Linking neighborhood social and built environments to children’s self-regulation

The small literature on self-regulation in neighborhood contexts supports a role for both the built environment and social environment in shaping components of child self-regulation. We build on a model of self-regulation, which highlights the bi-directional relations between more effortful aspects of self-regulation such as executive function and more automatic processes including stress physiology and emotion regulation (Blair and Ursache, 2011; Blair and Ku, 2022). Stress physiology involves biological regulation through the hypothalamic–pituitary–adrenal (HPA) axis, the parasympathetic nervous system (PNS), and the sympathetic nervous system (SNS; Holochwost et al., 2021). Automatic forms of attention emerge in infancy, and as children develop, more effortful forms of attention emerge, setting the stage for effortful aspects of emotion regulation and executive functions. Emotion regulation is defined as a set of contextually influenced, dynamic processes that modulate emotion (Eisenberg et al., 2004). Executive functions are cognitive skills that support goal-directed behavior through organizing, planning, and problem solving (Blair and Ursache, 2011).

Emerging work links multiple aspects of neighborhood context to children’s self-regulation, but much of this work has focused on the ways in which neighborhood risk factors undermine development of self-regulation with less attention to the possibility that neighborhood factors may also be promotive (Hyde et al., 2022). We highlight three pathways by which neighborhood context may confer risk for self-regulation as well as was the role of neighborhood protective factors in supporting self-regulation. A prominent pathway involves aspects of the neighborhood that impair self-regulation by directly affecting the brain and interconnected biological systems in a young child’s developing body. Neighborhood factors that operate through this pathway include increased exposure to toxicants (e.g., air pollution) or deprivation of access to the physical inputs necessary for healthy growth and development (e.g., nutrient dense food; Mikkelsen and Chehimi, 2007; Block et al., 2012; Jones et al., 2014; Bryant et al., 2020; Jackson et al., 2021).

Furthermore recent conceptualizations of early childhood social and cognitive adversity have considered risk along two dimensions—threat and deprivation—with specific predictions of how each type of risk may impact development (Sheridan and McLaughlin, 2016). Threats are defined as those aspects of the environment which may cause or can be perceived as potentially causing harm to children’s physical or psychological well-being. In the neighborhood context, possible threat exposures likely include crime, noise, physical disorder (e.g., dilapidated buildings), surveillance by police, and immigration raids. Exposure to threat can have direct impacts on children’s self-regulation. Threat leads to activation in stress response systems which over time can lead to dysregulation in these physiological systems, resulting in alterations to more automatic aspects of self-regulatory processes.

Deprivation involves children not receiving inputs that are necessary to support healthy growth and development. At the neighborhood level, children may experience deprivation because they do not receive adequate cognitive stimulation in neighborhoods that lack high quality educational resources, such as libraries, museums, childcare, or schools (Hyde et al., 2022). Exposure to deprivation is thought to more directly impact the most effortful aspects of self-regulation such as executive function by not providing the social or cognitive stimulation necessary to develop these higher level cognitive processes. Importantly, however, the integrative model of self-regulation describes how these automatic and effortful processes are linked and thus alterations to one component of self-regulation will likely lead to alterations in the other components as well (Blair and Ku, 2022).

Although less well studied, neighborhood level promotion of self-regulation may occur in the absence of risk factors or through the presence of protective factors—positive social and built environment resources—such as collective efficacy and greenspace. These protective factors may support self-regulation independently of neighborhood risk, or their effects may depend on the level of risk in the neighborhood. In line with the stress buffering hypothesis proposed by Luthar et al. (2000), these promotive factors may buffer children from the impacts of neighborhood risk factors on self-regulation by attenuating children’s experience of stress. Alternatively, however, the “overwhelming-risk” hypothesis (Luthar et al., 2000; Li et al., 2007) describes how high levels of risk can wash out the impact of protective factors.

### Social environment and self-regulation

#### Collective efficacy

Collective efficacy encompasses social cohesion (mutual trust among neighbors) and informal social control (willingness of neighbors to intervene in the service of shared goals; Sampson et al., 1997). Contexts of concentrated neighborhood disadvantage undermine the development of collective efficacy through limited opportunities for homeownership, decreased residential stability, and resource deprivation (Sampson et al., 1997). Importantly, although social cohesion is likely necessary for collective action through social control, it is not sufficient. The physical and psychological toll of resource deprivation can undermine the development of collective action even when personal connections are strong (Sampson et al., 1997). Several studies of child outcomes in the context of collective efficacy suggest that collective efficacy may impact children’s self-regulation. In children, collective efficacy has been shown to be related to better mental health (Xue et al., 2005) and behavior problems (Ingoldsby et al., 2006; Kohen et al., 2008). This relation between collective efficacy and both externalizing and internalizing behavior problems is evident as early as 3 years of age (Ma and Grogan-Kaylor, 2017). Other work has identified trajectories of neighborhood cohesion and their relation to mental health and behavior in adolescence (Kingsbury et al., 2015). Increasing neighborhood cohesion over the course of childhood was associated with lower hyperactivity and indirect aggression in adolescence. Growing up in neighborhoods low in social cohesion was associated with anxiety and depressive symptoms. Declines in neighborhood cohesion were also associated with hyperactivity. Neighborhood support has also been associated with parent perceptions of whether their school-aged children are flourishing as defined by curiosity about learning, resilience, and self-regulation (Kandasamy et al., 2018). Future work is needed to clarify whether collective efficacy impacts self-regulation specifically and whether these impacts are direct or mediated through home and school processes (detailed in the Mediating Pathways section below).

Furthermore, although not well studied in relation to children’s self-regulation, some evidence suggests that collective efficacy may be an important moderator of neighborhood risk factors (Hyde et al., 2022). For example, adolescents’ perceptions of social cohesion in their neighborhood moderated the association of neighborhood structural disadvantage (i.e., index of neighborhood characteristics) with adolescent depressive symptoms (Dawson et al., 2019). Without considering social cohesion as a moderator, neighborhood structural disadvantage was associated with greater depressive symptoms. Social cohesion, however, moderated this relation such that at high levels of social cohesion, higher neighborhood structural disadvantage was associated with lower depressive symptoms suggesting that at high levels of social cohesion, neighborhood structural disadvantage may not negatively impact adolescent depressive symptoms. Similarly, high neighborhood collective efficacy has been shown to buffer impacts of exposure to gun violence on adolescent’s functioning of corticolimbic circuits that support socioemotional processing (Gard et al., 2022). Positive social processes in the neighborhood have also been shown to buffer the association between neighborhood disadvantage and increased amygdala reactivity to threat (Suarez et al., 2022). More work is needed to understand the potential of collective efficacy to buffer the impact of social and built environment risks on children’s self-regulation.

#### Violence

A number of studies demonstrate that school-age children and adolescents living in historically disinvested neighborhoods experience serious forms of violence in their communities, which has broad negative impacts on multiple aspects of their health and development (Attar et al., 1994; Gorman-Smith and Tolan, 1998; Kliewer et al., 1998; Linares et al., 2001; Farver et al., 2005). Young children are also exposed to many different forms of neighborhood violence, including property crimes, assaults, and shootings (Taylor et al., 1994; Farver et al., 1999; Finkelhor et al., 2015). Theory suggests that early exposure to violence can shape multiple aspects of self-regulation including stress physiology, emotion regulation, and executive function (McCoy, 2013). Empirical work demonstrates that neighborhood violence shapes children’s attentional processes, and impulse control (Sharkey et al., 2012; McCoy et al., 2015b, 2016). In one study, children living in neighborhoods characterized by high levels of crime exhibited a more vigilant pattern of attention than children from lower-crime neighborhoods (McCoy et al., 2016). Similarly, living in close proximity to recent violent crime was associated with vigilant patterns of attention for children with low trait anxiety, whereas among highly anxious children, this exposure was associated with avoidant patterns of attention (McCoy et al., 2015b). Among preschool aged children, the occurrence of a homicide near their home in the past week was associated with lower levels of attention and impulse control as observed by research staff conducting one on one assessments with children in the school setting (Sharkey et al., 2012).

#### Surveillance by police

In the past 20 years, many police departments have adopted an approach of “proactive policing” which aims to deter criminal behavior through increasing police presence and stringently enforcing laws pertaining to minor crimes (Geller and Fagan, 2019; Justice, 2021). In neighborhoods targeted by these policing strategies, rates of nonconsensual “stop-question-frisk” police contact with individuals engaging in *legal* behaviors have greatly increased (Justice, 2021). Policing experienced in ethnic-racial minority and low-income neighborhoods may be more invasive and harmful than that in white middle-class neighborhoods (Shedd, 2015). For example, minors and young men of color are more likely to be subjected to stop, question, and frisk encounters (Fagan, 2017; Rudovsky and Harris, 2018). Although no work has examined whether these policing practices directly impact self-regulation in early childhood, one longitudinal study of adolescent girls demonstrated that self-control and responsibility declined following police contact (Hipwell et al., 2018). Furthermore, prior work has shown that involuntary police contact and the threat thereof is associated with psychological distress and academic achievement (Gottlieb and Wilson, 2019; Justice, 2021), suggesting a link to self-regulation through stress physiology that may be particularly strong for children of color, especially Black boys. Directly experiencing police stops has been related to greater psychological distress among adolescents, which in turn predicts lower academic grades (Del Toro et al., 2022). These associations were similar across racial ethnic groups, but rates of being stopped were higher among Black youth and boys. Vicarious police stops (e.g., knowing someone who has been stopped by the police or witnessing someone stopped by the police in the neighborhood or at school) also contribute to poorer health for Black and Latinx youth (but not for white youth) as well as lower academic outcomes for Black boys (Legewie and Fagan, 2019; Del Toro et al., 2022) and for Latinx adolescents (Del Toro et al., 2022). Black youth, however, have been shown to experience greater psychological distress than both white and Latinx youth following a vicarious stop, which in turn predicted poorer grades (Del Toro et al., 2022). Additionally, Black boys have been shown to be particularly impacted by the threat of police contact such that test scores declined because of surges in police surveillance in the neighborhood (Legewie and Fagan, 2019). Furthermore, adolescents with lower levels of self-control are more likely to be stopped by police and also experience greater intrusiveness from police and higher emotional distress during encounters (Jackson et al., 2020). Because of this greater experience of distress, these adolescents may then be at higher risk for even greater declines in self-regulatory skills.

#### Immigration raids

There has been an increased presence of Immigration and Customs Enforcement (ICE) agents in the U.S. interior since the creation of ICE as part of the Homeland Security Act (American Immigration Council, 2017). ICE activity is associated with greater reports of stress and lower self-rated health scores among adults in impacted communities (Williams et al., 2017). Children are also affected by ICE activity in their neighborhoods (Dreby, 2012; Lopez, 2019). Analyses of 115 drawings by children enduring anti-immigrant policies in Maricopa County, Phoenix, Arizona document young children’s preoccupations with the threat of family separation and the presence of ICE in their communities (Rodriguez Vega, 2018). This constant threat of familial separation and chronic uncertainty is theorized to have direct impacts on children both through deprivation and threat pathways (Barajas-Gonzalez et al., 2018, 2021). Although no studies have yet investigated direct impacts of immigration raids or ICE presence on child self-regulation, recent work has shown that immigration enforcement threat more generally is related to self-regulation in children in Pre-K (according to parent report), although it was unexpectedly related to higher ratings of self-regulation by independent observers (Barajas-Gonzalez et al., 2022b). This work suggests that neighborhood level factors such as ICE presence and raids—which likely increase immigration enforcement threat for children and families—may have impacts on children’s self-regulation skills.

### Built environment and self-regulation

#### Environmental pollutants

Exposure to environmental pollution comes from a number of sources which impact neighborhood air and water quality. Traffic related sources, including living near a major roadway or in an area of high traffic density, are a major cause of air pollution. Within urban areas, neighborhoods with higher percentages of families of color and families with lower socioeconomic status have greater exposure to air pollution (Hajat et al., 2013; Jones et al., 2014). Exposure to traffic related air pollution may lead to brain damage through neuroinflammation and oxidative stress pathways, or through neurotoxicity (Block et al., 2012). Areas of prefrontal cortex that support self-regulation may be particularly impacted by these neurobiological mechanisms (Calderón-Garcidueñas et al., 2008; Peterson et al., 2015), and air pollution has been shown to have direct effects on self-regulatory skills in childhood (Perera et al., 2012; Chiu et al., 2013; Sunyer et al., 2015; Harris et al., 2016). Living in high traffic density areas has been associated with teacher rated executive function skills in mid childhood (Harris et al., 2016). Exposure to traffic related air pollution has been associated with poorer inhibitory control (Chiu et al., 2013), slower growth in attention and working memory over a 12 month period (Sunyer et al., 2015), and higher attention problems (Perera et al., 2012).

Less work has examined pollution in drinking water which can be a conduit of other environmental exposures such as lead. Lead exposure in water is tied to neighborhood residence not only because of the age of housing stock and pipes, but also because of neighborhood struggles with financial resources, water sources, and aging infrastructure used for water distribution, as exemplified in the recent crisis in Flint, Michigan (Hanna-Attisha et al., 2016). Lead exposure has been associated with lower self-regulated attention among 4 and 5 year old children living in poverty (Davis et al., 2004).

#### Noise

High traffic density and having a major roadway nearby also create noise. Road traffic noise has been shown to increase stress (Babisch, 2011) and thus may have direct impacts on self-regulation by leading to repeated activation of, and thus alteration in, children’s physiological stress systems. Studies investigating relations of noise to self-regulation have found that traffic noise at school impacts attention measured by both neuropsychological tests (Van Kempen et al., 2010) and teacher observations (Forns et al., 2016). Furthermore, higher levels of road traffic noise has been linked to greater parent reported inattention among 8-year-old children (Weyde et al., 2017).

#### Food environment

Neighborhoods where low-income, minority, or immigrant families live have been shown to lack supermarkets, grocery stores, and farmers’ markets, resulting in lower availability of fresh, healthy, affordable foods (Mikkelsen and Chehimi, 2007). Low-income families also have to travel further than higher-income families in order to reach supermarkets (Mikkelsen and Chehimi, 2007). Some evidence suggests that fast-food restaurants may be more prevalent in low-income neighborhoods, but other work suggests comparable accessibility across high and low-income neighborhoods (see Mikkelsen and Chehimi, 2007 for a review). Even in the case of comparable accessibility, however, fast food may be more salient in low-income neighborhoods because of the scarcity of healthy alternatives (Mikkelsen and Chehimi, 2007). Access to and affordability of healthy foods as well as availability of unhealthy foods are dimensions of the neighborhood food environment that may play a role in the development of self-regulation through pathways related to child nutrition (detailed in the Mediating Pathways section below). Access to healthy foods such as fruits and vegetables provides nutrients that support children’s physical health and development (Morland et al., 2006; Powell et al., 2007; Bryant et al., 2020). One study to date has examined the direct link between the food environment and executive function among preschoolers (Bryant et al., 2020). Parent report of the food environment (access to, availability, and affordability of healthy food) was related to children’s executive function. Food environment measured at the census tract level (i.e., a dichotomous indicator of access to supermarkets, supercenters, and large grocery stores), however, was not related.

#### Greenspace

Exposure to natural features, such as water, grass, and trees varies greatly across urban environments and tends to be more limited in disinvested areas. There are three potential pathways through which green space may impact children’s self-regulation (Weeland et al., 2019). Access to greenspace may promote self-regulation by allowing children to experience more daylight and physical activity through playing outside (Beute and de Kort, 2014; Christian et al., 2015; Piepmeier et al., 2015). Natural greenspaces can also buffer against exposure to aspects of the built environment such as noise and pollution (Markevych et al., 2017; Weeland et al., 2019), and tree canopies have specifically been shown to be important for reducing traffic related air pollution and noise (Klingberg et al., 2017). Additionally, exposure to natural environments may replenish depleted self-regulatory resources (Kaplan and Berman, 2010) and restore affective and physiological processes that have been negatively impacted by stress (Ulrich, 1981, 1983; Ulrich et al., 1991). Two recent meta-analyses of the effect of exposure to nature among school-age children found benefits of nature for self-regulation across both correlational and quasi-experimental studies (Weeland et al., 2019). A few studies have specifically focused on the ways neighborhood greenspace relates to aspects of emotion regulation and executive function. In one study, the naturalness of the view from children’s homes was related to attention and inhibition for girls, but not for boys (Taylor et al., 2002). Green space around children’s homes longitudinally predicted self-regulatory problems reported by parents (Flouri et al., 2014). Higher lifelong residential neighborhood greenness longitudinally predicted better attention at 4–5 years of age and at 7 years of age (Dadvand et al., 2017). Greenness surrounding children’s neighborhood, including home, commute, and school was associated with greater increases in working memory and declines in inattentiveness over a 12-month period among school-aged children (Dadvand et al., 2015).

#### Physical disorder

Physical disorder includes evidence of deterioration in the neighborhood, such as graffiti, litter, abandoned cars, broken windows, abandoned, vandalized or run-down buildings, and vacant housing (Sampson and Raudenbush, 1999). Aspects of physical disorder are visually prominent cues which can indicate crime in the neighborhood (Sampson and Raudenbush, 1999) and make residents feel fearful (Garvin et al., 2013). Additionally, disorder may indicate low collective efficacy for improving neighborhood conditions (Sampson and Raudenbush, 1999) in part because fear and related social isolation can impede collective efficacy, which perpetuates further disorder (Garvin et al., 2013). Furthermore, individuals living in areas with physical disorder indicate feeling negative emotions such as sadness, depression, and anxiety as well as feelings of being neglected, and physical disorder has been linked to mental health burden (Garvin et al., 2013). Consistent with these feelings of fear and negative emotionality, theoretical perspectives suggest that physical disorder promotes chronic stress and changes in physiological stress responses (Hill et al., 2005; Garvin et al., 2013). To our knowledge, studies have not yet examined the link between physical disorder and children’s self-regulation, but we hypothesize that it may affect children directly as it does adults or indirectly *via* the home environment (described in the mediation pathways section below).

#### Education related resources

Education related resources, such as day care centers, schools, museums, libraries, community centers, and higher education institutions greatly influence children’s opportunities for learning, and the presence and quality of these resources varies across neighborhoods even when they are similar in terms of socioeconomic characteristics (Wei W.S. et al., 2021). Access to high quality learning environments may support interactions with caregivers and teachers that could promote child self-regulation development (Rimm-Kaufman et al., 2009; Ursache et al., 2012; Wei W.S. et al., 2021). For example, higher quality child care has been shown to predict better executive function skills (Berry et al., 2014). Furthermore, access to education resources such as libraries and museums may scaffold parents in book sharing and play, which can improve the parent–child relationship (Weisleder et al., 2019) and thus self-regulation (as described in the section on home environment and resources pathway). Not having access to these resources may be a form of deprivation (i.e., limited cognitive and social stimulation), which is theorized to have direct consequences for executive function development (Sheridan and McLaughlin, 2016; Sheridan et al., 2017). Few studies have directly examined neighborhood education related resources in relation to self-regulation development in early childhood. One study investigated the relation of neighborhood resources more broadly to children’s gains in executive functioning over the pre-K year, but did not find any evidence of associations (Wei W.S. et al., 2021).

## Mediating pathways from neighborhood to self-regulation

In examining how neighborhood context influences self-regulation, we propose three mediating pathways across multiple ecological levels: (1) classroom environment and resources, (2) home environment and resources, and (3) child health behaviors. Although few studies have explicitly tested these mediating pathways, prior work links each of these potential mediators to both neighborhood context and to children’s self-regulation. The studies reviewed below are not a comprehensive representation of this work but rather salient examples that suggest support for these pathways. Furthermore, the review emphasizes the emerging evidence linking neighborhood environment to the mediators given robust literatures that have established links from the mediators to self-regulation. In line with the bioecological model of human development (Bronfenbrenner and Morris, 2006; Center for Child and Family Well-Being, 2021), child health behaviors are nested within home environment and resources, which are nested within classroom environment and resources. Although beyond the scope of this review, empirical work has supported the theory that these levels reciprocally influence each other – context shapes child behavior, and child behavior shapes home and school contexts (Goldstein et al., 2001; Meltzer and Mindell, 2007; Aizer, 2008; Bell and Belsky, 2008; Pettit and Arsiwalla, 2008; Huang et al., 2013; Paschall and Mastergeorge, 2016; Brusseau and Burns, 2018; McKinnon and Blair, 2019; Pakarinen et al., 2021).

### Classroom environment and resources

Neighborhood disinvestment is associated with characteristics of classrooms that provide early childhood care and learning opportunities. Broadly, disinvested neighborhoods tend to have public schools that are characterized by low resources, lower graduation rates, lower teacher certifications, and dilapidated facilities (Rothstein, 2013; Lynch, 2017; Pruitt et al., 2019). Neighborhood structural disadvantage has also been associated with lower quality of community child care programs (Phillips et al., 1994; Fuller et al., 1997; Loeb et al., 2004; Burchinal et al., 2008) although public investment in high quality early education may mean that there is a somewhat higher prevalence of high quality programs in some of the most disadvantaged neighborhoods (Phillips et al., 1994; Fuller et al., 2003). Neighborhood collective efficacy, however, may theoretically promote high quality classrooms by helping teachers to manage their own stressors as evidence demonstrates a link between neighborhood social resources and social support among residents (Mair et al., 2021). Early childhood classrooms can in turn support children’s self-regulatory skills both through providing activities that explicitly teach emotion regulation and executive function skills as well as through creating safe, nurturing, and predictable environments that allow children to experience a level of physiological stress arousal that is conducive to using and developing emotion regulation and executive function skills (Ursache et al., 2012; Raver, 2014). Although there is nuance across studies, there evidence that higher quality classrooms are associated with higher self-regulatory skills for children from toddlerhood through kindergarten (Rimm-Kaufman et al., 2009; Weiland et al., 2013; Salminen et al., 2021). To date, only one study has directly examined classroom environment and resources as mediators of the link between neighborhood characteristics and children’s self-regulation skills. This study found that lower neighborhood socioeconomic status was associated with lower classroom instructional quality which in turn was associated with smaller increases in children’s executive function skills over the pre-K year (Wei W.S. et al., 2021). Relatedly, the link between neighborhood poverty and children’s social–emotional skills more generally has been shown to be mediated by emotional support in the classroom (McCoy et al., 2015a), suggesting a similar pathway for classroom emotional support and self-regulation specifically.

### Home environment and resources

Two models describe the home environment and resources as a link between neighborhoods and child outcomes: the family stress model (McLoyd, 1990; Conger et al., 1992) and the family investment model (Shuey and Leventhal, 2017). In line with the family stress model, parents’ exposure to neighborhood stressors affects their own well-being and their parenting behaviors in ways that relate to children’s development (Garner et al., 2021). Multiple aspects of neighborhood disadvantage can directly increase the stress that parents navigate on a daily basis, inevitably affecting their ability to provide the emotionally supportive, safe, nurturing, cognitively stimulating, and predictable home environments that are necessary to support self-regulation development (Brown and Ackerman, 2011; McCoy, 2013; Minh et al., 2017). For example, neighborhood violence has been shown to make parents less available for physical and emotional caretaking (Margolin, 1998; Farver et al., 2005). Greater neighborhood disorder is also associated with greater family conflict and harsher parenting (Barajas-Gonzalez and Brooks-Gunn, 2014). Neighborhood factors, however, can also help to buffer parents from these stressors. For example, collective efficacy is associated with greater social support among residents which likely promotes parent well-being and quality of parenting both directly and through buffering parents from stressful experiences (Armstrong et al., 2005; Mair et al., 2021). Similarly, neighborhood social cohesion has been shown to buffer the impact of financial deprivation on adult psychological distress such that among adults experiencing financial deprivation, those whose neighborhoods had high social cohesion experienced less psychological distress than those whose neighborhoods had low social cohesion (Erdem et al., 2016).

The family investment model suggests that neighborhood affluence increases parents’ access to resources that support children’s cognitive development and can reinforce effective parenting behaviors (Shuey and Leventhal, 2017). For example, neighborhoods that offer access to resources such as museums and libraries likely support parents to engage with their children in cognitively stimulating ways. More recently, there has been increasing recognition that these pathways are intertwined as they relate to child development (Weisleder et al., 2019; Garner et al., 2021). The relational health framework encompasses both of these perspectives by focusing on how safe, cognitively stimulating, emotionally responsive, and stable relationships can buffer children’s adverse experiences and promote healthy development (Garner et al., 2021). Importantly, neighborhoods that are safe, stable, and nurturing communities include access to resources and services that promote the development of early relational health for families and their children (Garner et al., 2021). In turn these aspects of early relational health—emotionally responsive parenting behaviors and engagement in cognitively stimulating parent–child activities—have been shown to buffer child stress and support the development of self-regulation (Blair et al., 2014; Thompson, 2015; Weisleder et al., 2019; Hyde et al., 2022; Piccolo et al., 2022).

### Child health behaviors

Third, the built and social environments that characterize children’s neighborhoods influence children’s health behaviors, including sleep, physical activity, and nutrition. Sleep, physical activity, and nutrition in turn play a role in setting the stage for the development of self-regulation skills (Becker et al., 2014; Williams et al., 2017; Breitenstein et al., 2021; Jackson et al., 2021).

#### Sleep

Neighborhood noise, environmental pollutants, access to physical activity amenities, population density, violence, and safety concerns have been shown to increase risk for poor sleep health and sleep disorders among children (Meldrum and Restivo, 2014; Lawrence et al., 2018; Hale et al., 2019; Koinis-Mitchell et al., 2019; Philbrook et al., 2020). Both theoretical and empirical research demonstrate that multiple aspects of sleep play an important role in children’s development of multiple aspects of self-regulation (Williams et al., 2017; Breitenstein et al., 2021). A conceptual framework proposed by Breitenstein and colleagues suggests that in early childhood, sleep is reciprocally related to both physiological stress systems and to functioning of the prefrontal cortex and anterior cingulate cortex, brain areas that support self-regulation (Breitenstein et al., 2021). Consistent with this theory, sleep behavior, which undergoes rapid development in early childhood (Acebo et al., 2005), has been shown to longitudinally predict emotional and attentional aspects of self-regulation (Williams et al., 2017). Furthermore, sleep duration is consistently related to executive function both cross-sectionally and longitudinally (see Breitenstein et al., 2021 for a review).

#### Physical activity

Neighborhood housing density, walkability, traffic speed/volume, vegetation (ie., presence of street trees), access to recreation facilities, land use mix, and disorder, are all consistent predictors of physical activity among children (Roemmich et al., 2006; Miles, 2008; Ding et al., 2011). The role of park access in promoting physical activity is less consistent with some evidence suggesting null or inverse associations (Ding et al., 2011; McGrath et al., 2015). These unexpected findings may be explained by work demonstrating that physical disorder and perceived safety play an important role in whether adults encourage children to use local playgrounds (Miles, 2008). Work on the role of crime and safety, however, has been limited and more high quality work is needed to investigate this association (Ding et al., 2011), especially because for Black families, perceived neighborhood crime may be a particularly important factor in children’s sedentary behavior (Budd et al., 2015). Research with both animals and humans, has shown that physical activity enhances aspects of self-regulation such as inhibitory control and executive function (Becker et al., 2014). For example, among pre-kindergarten children, greater time spent in active play during recess was related to greater self-regulation (Becker et al., 2014) and engaging in at least 60 min of physical activity 7 days a week was associated with self-regulation in middle childhood (López-Gil et al., 2020). Furthermore, experimental work which implemented an exercise intervention with overweight 7–11 year old children improved executive function and increased activity in the prefrontal cortex, an area of the brain important for self-regulation (Davis et al., 2011).

#### Nutrition

The relation between neighborhood access to healthy foods and individual consumption of fruits and vegetables has mainly been studied in adults, but this pathway likely extends to children (Mikkelsen and Chehimi, 2007). Furthermore, Latinx mothers have described how pervasive access to fast food restaurants and intensive marketing to children have led their children to consume more unhealthy foods (Jones, 2002). Nutrients from healthy foods such as fruits and vegetables are necessary to support brain development underlying cognition, and nutritional deficiencies may have greater impacts on cognition in times of rapid brain development such as in early childhood (Morland et al., 2006; Powell et al., 2007; Nyaradi et al., 2013; Wachs et al., 2014; UDHHS, 2015; Bryant et al., 2020). Consistent with this pathway, food insecurity—low or uncertain access to affordable nutritious foods—has been associated with increased risk for self-regulation difficulties in early childhood (Jackson et al., 2021).

Thus, theory and empirical research support three multilevel mediation pathways linking neighborhood context to children’s self-regulation through (1) classroom environment and resources, (2) home environment and resources, and (3) child health behaviors. Future work is needed to explicitly test these mediation pathways individually and in parallel.

## Cultural strengths

Families of color have particular cultural strengths that are often absent from discussion about disinvested neighborhoods. For example, some of the cultural assets of Black American families include optimism, extended kin and social networks, and religiosity and spirituality (Harrell, 2022; Lloyd et al., 2022). Similarly, some of the cultural assets of Latinx families include religiosity and spirituality, a collective orientation, and familism, which emphasizes solidarity, loyalty and reciprocity among family members (Zambrana and Zoppi, 2002; Calzada et al., 2013). Theoretically, these cultural assets can help buffer the impact of neighborhood risk factors on child outcomes by shaping processes at multiple levels and at multiple points in the pathway from neighborhood to self-regulation. We explore collective efficacy and home environment as two examples of pathways through which cultural assets may support self-regulation in the face of neighborhood disadvantage. We focus on Black and Latinx families who are better represented in the literature to date, but emerging work is also investigating these relations in Asian American families (Wei W. et al., 2021).

Cultural assets may scaffold the development of collective efficacy. For example, one study showed that higher concentrations of African American and residentially stable residents in one’s neighborhood was associated with greater cumulative social support and perceptions of neighborhood cohesion which in turn was linked to fewer internalizing symptoms among adolescents (Hurd et al., 2013). Furthermore, Black churches, which are a central institution in urban Black neighborhoods, can support collective efficacy by coordinating residents and organizations to address youth violence (Pegram et al., 2016).

Additionally, cultural strengths may buffer the link between neighborhood risk factors and children’s self-regulation through processes in the home environment. For example, in a cross-sectional study of Mexican American youth, higher levels of familism behaviors were protective for youth resilience at both low and high levels of neighborhood hazards (i.e., crime, gangs, traffic, and noise; Romero et al., 2020). Similarly, the association between perceptions of neighborhood danger and harsh parenting has been shown to be moderated by cultural values among Mexican American families (Gonzales et al., 2011; White and Roosa, 2012). Furthermore, cultural strengths can support racial-ethnic socialization (Rivas-Drake and Witherspoon, 2013; Brittian Loyd and Williams, 2017; Anderson and Stevenson, 2019). Research with adolescents suggests that racial-ethnic socialization has potential to buffer impacts of stressful discriminatory experiences on self-regulation (Rivas-Drake et al., 2014; Umaña-Taylor and Rivas-Drake, 2021), but whether these findings generalize to neighborhood level stressors or to young children has not yet been explored.

## Evidence from interventions in high risk neighborhoods

The conceptual model elucidates multiple points for intervention to address neighborhood impacts on child self-regulation. First, we highlight promising approaches that directly change the built or social environment. Next, we summarize key findings from prevention science, which has focused primarily on mitigating the impact of poverty through classroom and home-level interventions. Taken together, experimental evidence strengthens confidence in the causal nature of the links in Figure 1, supports the value of interventions to protect children from neighborhood risk, and underscores the need for structural solutions to structural problems (Brown et al., 2019).

### Neighborhood-level intervention and policy change

With increasing understanding of structural racism, there is increasing commitment to addressing the problem directly by re-investing in neighborhoods. Bailey et al. (2017) describe how *place-based, multisector, equity initiatives* can work. Federal initiatives launched in the past decade (Promise Neighborhoods and Choice Neighborhoods) are based largely on the success of Purpose Built Communities (2015) and their original 1995 redevelopment initiative in Atlanta, GA. In addition to $123 million (compared to no capital investment in the 30 years prior), this partnership between the Atlanta Housing Authority, leaders from the community and public housing, and philanthropy resulted in high-quality, mixed-income housing; cradle-to-college education, and a series of programs chosen by residents – with dramatic changes in employment and crime within 10 years (Bailey et al., 2017).

Specific *improvements to the built environment* in disinvested neighborhoods have also been evaluated. A review of experimental and quasi-experimental studies indicates that housing and blight remediation of buildings and land is practical, sustainable, and shows consistent reductions in violent crime (Kondo et al., 2018). For example, a citywide cluster randomized trial in Philadelphia found that “cleaning and greening” vacant lots significantly reduced crime and gun violence in particular; further, based on reports from residents near greened lots, this low-cost remediation increased use of outdoor spaces for relaxing and socializing and reduced fear (Branas et al., 2018), feelings of worthlessness, and depression (South et al., 2018).

*Participatory budgeting* is a “democratic process in which community members decide how to spend part of a public budget.” Originating as an anti-poverty policy in Brazil, it is now used broadly to allocate state and local budgets with important neighborhood impacts. Municipal governments with participatory budgeting (120 of Brazil’s largest cities) allocated more funds to sanitation and health services than other municipalities (adjusting for economic and political differences). Strikingly, in cities with sustained political commitment for participatory budgeting for >8 years, records revealed a nearly 20% drop in infant mortality (Gonçalves, 2014; Touchton and Wampler, 2014).

*Public investment in high quality early care and education* is seen as a powerful policy lever to address inequities rooted in structural racism (Heckman et al., 2013). Nearly all states are building the infrastructure to provide high-quality preschool, reaching 30% of 4-year-old across the country (Phillips et al., 2017). Increased access to educational opportunities lays the foundation for classroom environments which may buffer neighborhood risk. For example, Boston’s pre-K program (with evidence-based curricula, bachelors/masters-level teachers, and coaching) improved executive functioning and emotional development (as well as literacy and math; Weiland and Yoshikawa, 2013). Ongoing research evaluates impacts at scale (Phillips et al., 2017) and scholars advocate for elevating the status of the early education workforce, which is majority women of color, often not earning a living wage (U.S. Department of Education, 2016; Washington, 2017).

### Classroom and home-level intervention

Robust evidence documents the central role of classroom and home environments in promoting child self-regulation within high risk contexts. Key findings from this literature align with our conceptualization: (1) there is substantial variability in children’s emerging self-regulation within historically disinvested neighborhoods; (2) home and classroom environments are malleable; and (3) bolstering adult capacity to provide emotionally responsive, cognitively stimulating environments despite the broader context confers meaningful benefits for child self-regulation (e.g., Head Start REDI; Bierman et al., 2008; Chicago School Readiness Project; Raver et al., 2009; Family Check-up in WIC clinics; Chang et al., 2014; Video Interaction Project in primary care; Weisleder et al., 2016; Canfield et al., 2020). For example, in Head Start centers in historically disinvested neighborhoods in Chicago, improvements in teacher-child relationships explained improvements in self-regulation (which explained academic outcomes Raver et al., 2011; Jones et al., 2013). Long-term follow-up considered the ongoing influence of crime (average > 600 incidents/year); though modest, intervention impact on social–emotional trajectories was actually stronger for children who attended elementary schools in census tracts with higher levels of crime (McCoy et al., 2018).

ParentCorps is a family-centered enhancement to pre-Kindergarten in historically disinvested neighborhoods and includes home and classroom components, as well as a child component which directly supports healthy eating, activity, and emotion regulation (Dawson-McClure et al., 2022). Trials in New York City schools demonstrate that ParentCorps is working as intended, and that changes in children’s proximal environments during this critical developmental period lead to sustained improvements across three domains: academic achievement, physical and mental health (Brotman et al., 2011, 2012, 2013, 2016; Dawson-McClure et al., 2015). While ParentCorps was not designed to address neighborhood-level factors, fundamental to this approach is building relationships with and a sense of community among parents – an enduring “corps” of support (Brotman et al., 2011). Facilitators explicitly affirm parents’ inherent value, actively support their autonomy, and honor culture as important and adaptive (Garcia-Coll et al., 1996). In this context of being seen, heard, and cared for – parents share about their lived racial experiences, reflect on sources of support in their community, and consider changes in alignment with their values and beliefs (Dawson-McClure et al., 2022).

## Limitations

Despite the many innovative aspects of this model conceptualizing the development of early childhood self-regulation in neighborhood context, some important limitations remain. First, the model focuses on self-regulation in early childhood. Extending this model to adolescence, a second period of heightened developmental plasticity in brain areas important for self-regulation, is important and will necessitate consideration of the increasing ways in which older children engage with their neighborhood contexts. Different mediational processes, for example, through experiences with peers and through racial-ethnic socialization also warrant consideration. Second, a life-span model will also need to consider how the impact of neighborhood factors may change with child age and consider issues of timing between neighborhood exposures and self-regulatory outcomes. Relatedly, this model does not consider genetic levels of influence or the ways in which intergenerational transmission of self-regulation may occur in the context of historical disinvestment. Third, individual-level factors that may moderate relations between neighborhood context and self-regulation are not a focus of this model. For example, individual-level factors may preclude a family from accessing neighborhood resources because of legal restrictions or because they may not feel safe or welcome doing so (Barajas-Gonzalez et al., 2022a). Finally, the model situates the development of self-regulation within structural racism, but does not discuss the ways in which *interpersonal* racism and discrimination may affect self-regulation. Recent work demonstrates that racism is a ubiquitous experience for children of color across many different types of neighborhoods (Zimmerman et al., 2022), and future work is needed to conceptualize and examine the ways in which interpersonal racism and discrimination impact self-regulation in these contexts.

## Conclusion

Our conceptual model of self-regulation in the context of historically disinvested neighborhoods advances understanding of the ways in which built and social environments shape the development of self-regulation in early childhood. In centering children of color growing up in historically disinvested neighborhoods, this model takes as its starting point the ways in which structures of racism and social stratification have shaped the built and social environment. Furthermore, we advance a multilevel approach which examines classroom environment and resources, home environment and resources, and child health as three potential pathways linking neighborhood contexts to self-regulation. Finally, racial-ethnic cultural strengths and multilevel interventions have the potential to buffer children’s development of self-regulation in disinvested neighborhood contexts. Advancing multilevel approaches to understand the development of self-regulation among children of color living in historically disinvested neighborhoods is an important step in efforts to promote equity in health and education.

## Author contributions

All authors listed have made a substantial, direct, and intellectual contribution to the work and approved it for publication.

## Funding

Support for this research was provided by the National Heart Lung and Blood Institute grant K01HL138114.

## Conflict of interest

The authors declare that the research was conducted in the absence of any commercial or financial relationships that could be construed as a potential conflict of interest.

## Publisher’s note

All claims expressed in this article are solely those of the authors and do not necessarily represent those of their affiliated organizations, or those of the publisher, the editors and the reviewers. Any product that may be evaluated in this article, or claim that may be made by its manufacturer, is not guaranteed or endorsed by the publisher.
